# Association Between Perioperative Hypothermia and Surgical Site Infection After Elective Abdominal Surgery: A Prospective Cohort Study

**DOI:** 10.7759/cureus.11145

**Published:** 2020-10-25

**Authors:** Tayyab Siddiqiui, K.M. Inam Pal, Fatima Shaukat, Hadika Mubashir, Alishah Akbar Ali, Muhammad Jehangir A Malik, Noman Shahzad

**Affiliations:** 1 Surgery, Aga Khan University, Karachi, PAK; 2 Oncology, Aga Khan University, Karachi, PAK; 3 Surgery, Northern Lincolnshire and Goole NHS Foundation Trust, Scunthorpe, GBR

**Keywords:** hypothermia, normothermia, surgical site infection

## Abstract

Introduction

Surgical site infections (SSIs) account for 14-16% of nosocomial infections and are one of the major causes of increased morbidity, hospital stay, cost of care, and even mortality. Hypothermia as a risk factor for SSI is debated but there is lack of conclusive evidence. The present study explores the association of hypothermia with SSI.

Methodology

This is a prospective cohort study conducted on adult patients who underwent elective laparotomy. Patients were divided into two cohorts, the Hypothermia Cohort and the Normothermia Cohort, based upon episodes of hypothermia of <36^0^C in the perioperative period. SSI was diagnosed based upon criteria defined by the Center for Disease Control and Prevention (CDC). Postoperative follow-up to detect SSI was done until 30 days after the operation.

Results

A total of 183 patients met the selection criteria and were included in the study. Ninety patients (49%) had perioperative hypothermia and were followed in the Hypothermia Cohort, while 93 patients (51%) who remained normothermic in the perioperative period were followed in the Normothermia Cohort. Mean age of the patients was 49.77 +/- 14.82 years. Almost two-thirds of the participants were females (63.9%). Patients who developed hypothermia were significantly older and had lower BMI. Also the proportion of female patients was significantly higher in the Normothermic Cohort.

Rate of SSI was similar in both groups (10% versus 10.8%) with p-value of 0.867. Multivariable regression analysis also failed to show any significant association between hypothermia and SSI.

Conclusion

Our study failed to show any statistically significant association between hypothermia and surgical site infection.

## Introduction

Surgical site infections (SSIs) account for 14-16% of nosocomial infections and are one of the major causes of increased morbidity, hospital stay, cost of care, and even mortality [1]. Known risk factors of SSIs include extremes of age, cigarette smoking, poor nutritional status, obesity, duration of surgery, poor glycemic control, and poor surgical techniques [1-5]. Homeostasis in the perioperative period is associated with favourable postoperative outcomes and maintenance of normothermia is recommended by safe surgery guidelines of the World Health Organization (WHO) and Center for Disease Control and Prevention (CDC) but there is lack of conclusive evidence on its impact on SSIs [1,6,7]. A number of studies have shown that perioperative hypothermia below 36^o^C is linked to increased risk of SSIs [8]. It is reported that with every 1^o^C fall in temperature below normal (36^o^C) in perioperative period, risk of SSI increases three times [8,9].

Impairment of neutrophilic function, peripheral vasoconstriction leading to hypoxia, malfunction of the humoral defense system, and high metabolic demand are possible ways hypothermia potentially impacts immunity [8,10].

On the other hand, no association of SSIs with hypothermia was found in other studies [1,11].

The present study explores the association of SSI with hypothermia on prospectively collected data.

Objective

To determine relative risk of surgical site infection in patients who developed hypothermia compared to those who did not develop hypothermia in the perioperative period, in patients undergoing elective abdominal surgery.

## Materials and methods

We conducted a prospective cohort study at Aga Khan University Hospital in Karachi, Pakistan. All adult patients above 18 years of age who underwent elective laparotomy were eligible for inclusion in the study. These laparotomies could be for general surgical, urological, or gynaecological problems. We excluded patients whose duration of operation was more than three hours. Also, patients who declined to participate were excluded. Body temperature was monitored preoperatively, intra-operatively, and postoperatively in the recovery unit as is routine. Based upon any episode of hypothermia of <36^0^C in the perioperative period, patients were divided into two groups. One group comprised patients who had an episode of hypothermia in the perioperative period (Hypothermia Cohort) while the other group included patients who did not have hypothermia (Normothermia Cohort). Postoperative follow-up to detect surgical site infection was done until 30 days after operation.

Data was collected with a specifically designed questionnaire. Perioperative hypothermia was defined as body temperature below 36^0^C in pre-, intra- or postoperative phase. Diagnosis of surgical site infection was made based upon criteria defined by the CDC which includes purulent discharge, positive culture, or clinical diagnosis by attending surgeon within 30 days of operation. Data were collected on potential confounding variables including age, gender, duration of operation, co-morbidities, and American Association of Anaesthesia (ASA) level.

Sample size was calculated using WHO software for sample size calculation. Keeping frequency of SSIs as 19% in patients who developed perioperative hypothermia and 6% in patients who did not get hypothermia as reported by Lehtinen et al. [1], with power of 80% and level of significance of 5%, a sample of at least 80 was required in each group. With anticipated loss to follow-up of 10%, we aimed at 88 patients in each group leading to the total patients required in our study to be at least 176.

Duration of study

The study duration was October 1, 2017, to December 31, 2017

Ethics approval

Approval was obtained from Ethics Review Committee (ERC) of The Aga Khan University Hospital with approval number of 4940-Sur-ERC-17.

Data collection process

All patients who underwent laparotomy and met selection criteria were enrolled in the study. Data was collected in an anonymized form to maintain confidentiality.

Data analysis

Data was entered and analyzed using Statistical Package for Social Sciences (SPSS) version 20 (IBM Corp., Armonk, NY, USA). Quantitative variables like age and duration of surgery are described in terms of means with standard deviations. Qualitative variables like gender, hypertension (HTN), diabetes mellitus (DM), American Association of Anesthesiologists (ASA) level, and SSI are reported in terms of frequencies and percentages. Comparability of co-variates between two cohorts is assessed using either chi-square test or Fischer exact test for categorical variables and independent sample t-test for continuous variables. Comparison of SSI between two cohorts was done by using chi-square test. All potential confounders like age, duration of surgery, DM, HTN, gender, and ASA level were checked and adjusted when needed using multivariable logistic regression analysis. A p-value of less than 0.05 is considered significant.

## Results

A total of 309 laparotomies were performed from October to December 2017. Out of these 183 patients met the selection criteria and were included in the study. Of those included in the study 49% (90) had perioperative hypothermia and were followed in the Hypothermia Cohort, while 51% (93) remained normothermic in the perioperative period and were followed in the Normothermia Cohort. Mean age of the patients was 49.77 +/- 14.82 years. Almost two-thirds of the participants were females (63.9%). Diabetes and hypertension were present in 22.4% and 36.6% of patients, respectively. Active smokers were 24 (13.1%). Almost all the patients (except three) received pre-operative prophylactic antibiotics. Mean BMI of the patients was 27.33 +/- 5.75 kg/m^2^.

Out of 183 patients, 17 patients (9.3%) were ASA I, 126 (68.9%) were ASA II, 37 patients (20.2%) were ASA III, and three patients (1.6%) were ASA IV. Most of the patients (122/66.7%) had laparotomy for general surgical causes, followed by gynecological cases (55/30%) and only six (3.3%) urological surgeries. Mean duration of surgery was 118.78 +/- 48.46 minutes.

Overall 19 (10.4%) patients developed surgical site infection.

Our data showed that patients who developed hypothermia were significantly older and had lower BMI than those who remained normothermic. Also the proportion of female patients was significantly higher in the Normothermic Cohort. The proportion of general surgical procedures was higher in the Hypothermic Cohort and so was the duration of operation. Details of distributions of patients’ demographics and underlying risk factors for SSIs among two cohorts are given in Table 1.

**Table 1:** Characteristics of the two cohorts ASA: American Society of Anesthesiologists, BMI: body mass index, SD: standard deviation

Variable		Hypothermia (N = 90) n (%)	Normothermia (N = 93) n (%)	P Value
Age (Years)		52.87 +/- 15.46	46.76 +/- 13.59	0.005
Gender (Male)		45 (50)	21 (22.6)	<0.001
Diabetes Mellitus		19 (21.1)	22 (23.7)	0.680
Hypertension		33 (36.7)	34 (36.6)	0.988
Smoking		15 (16.7)	9 (9.7)	0.161
Blood Transfusion		1 (1.1)	1 (1.1)	0.743
Use to Steroids		2 (2.2)	1 (1.1)	0.488
Prophylactic Antibiotics		89 (98.9)	91 (97.8)	0.512
ASA level (%)	ASA I	10 (11.1)	7 (7.5)	0.293
	ASA II	63 (70)	63 (67.7)	
	ASA III	17 (18.9)	20 (21.5)	
	ASA IV	0	3 (3.2)	
Types of surgery	General Surgery	69 (76.7)	53 (57.0)	0.001
	Gynecology	16 (17.8)	39 (41.9)	
	Urology	5 (5.6)	1 (1.1)	
Duration of Surgery in min (SD)		136.59 +/- 49.3	101.55 +/- 41.03	<0.001
BMI (SD)		25 +/- 5.02	28.62 +/- 6.13	0.002

Rate of SSI was similar in hypothermia and non-hypothermia groups (10% versus 10.8%) with a p-value of 0.867. Multivariable logistic regression analysis was performed to adjust for age, gender, BMI, duration of surgery, and type of surgery as these had significantly different distribution among the two cohorts. Adjusted analysis also failed to show any significant association between hypothermia and surgical site infection.

## Discussion

Maintaining normothermia during the perioperative period is associated with better surgical outcomes. Despite various measures taken to prevent hypothermia in patients, the perioperative period remains a vulnerable phase when patients are at greater risk of becoming hypothermic. Surgical site infection is one of the adverse outcomes linked to episodes of hypothermia. Our study has failed to demonstrate a statistically significant association between hypothermia and surgical site infection. These findings are consistent with findings of Lehtinen et al. and Baucom et al. who conducted a case-control study and a cohort study, respectively, and failed to show any association of hypothermia and SSI [1,11]. On the contrary, a randomised controlled trial conducted by Kurtz et al. showed that the incidence of surgical site infection is increased from 6% in normothermic patients to 19% in those who develop hypothermia [12]. 

Though our data did not show any significant association, our sample size was not adequate to detect smaller differences in risk of surgical site infection. Furthermore, we did not measure severity of hypothermia, which could be a factor in putting some patients at greater risk than others. This needs to be explored further in future studies.

## Conclusions

Our study failed to show statistically significant association between hypothermia and surgical site infection. Lack of power could be one reason as higher power is needed to detect smaller differences. We recommend that further studies powered to detect smaller differences should be conducted.
